# Hope influences learning engagement through generative AI acceptance among Chinese college students: conditional direct effect of growth mindset

**DOI:** 10.3389/fpsyg.2026.1842826

**Published:** 2026-07-17

**Authors:** Chao Deng, Yinshun Zhang, Xin Liao

**Affiliations:** 1 School of Computer Science and Artificial Intelligence (Industrial Software), Guangdong University of Science and Technology, Dongguan, Guangdong, China; 2 School of Financial Technology and Accounting, Guangdong University of Science and Technology, Dongguan, Guangdong, China; 3 Department of Convergence Lifelong Education, Hanseo University, Seosan, Republic of Korea; 4 Computer Science and Technology, Guangdong University of Technology, Guangzhou, China

**Keywords:** Chinese college student, conditional direct effect, generative AI acceptance, growth mindset, hope, learning engagement

## Abstract

College students' learning engagement is widely regarded as an important indicator of learning quality and academic development. Understanding the psychological and technological factors associated with learning engagement has become increasingly important in AI-supported learning environments. This study examined the relationships among hope, generative AI acceptance (GAA), growth mindset (GM), and learning engagement (LE) by proposing and testing a conditional direct effect model. Data were collected from 478 Chinese college students through an online questionnaire survey. SPSS 26.0, PROCESS 4.2, and AMOS 24.0 were used to conduct descriptive statistics, reliability and validity analyses, confirmatory factor analysis, correlation analysis, and conditional direct effect analysis. The results indicated that hope was positively associated with learning engagement. Generative AI acceptance partially mediated the relationship between hope and learning engagement. In addition, growth mindset significantly moderated the direct association between hope and learning engagement. Specifically, the positive association between hope and learning engagement was stronger among students with lower levels of growth mindset and weaker among those with higher levels of growth mindset, suggesting a compensatory pattern between these two psychological resources. The findings highlight the complementary roles of psychological resources and technology acceptance in understanding learning engagement in AI-supported learning environments. This study contributes to the literature by integrating hope, generative AI acceptance, and growth mindset within a single framework and provides empirical evidence regarding the psychological and technological factors associated with learning engagement among college students. The findings also offer practical implications for higher education institutions seeking to support student engagement in the generative AI era.

## 1 Introduction

Learning engagement is generally considered to be an essential variable for assessing learning quality and process. Fredricks et al. (2004) argued that learning engagement consists of multiple dimensions, and they emphasized that learning engagement consists of behavioral, affective, and cognitive engagements. Since then, many researchers have confirmed that this three-dimensional model is universal and applicable to various levels of learning (Lam et al., 2012, 2014; Wang et al., 2019). Given its close association with academic achievement, persistence, and learning experiences, learning engagement has become a central topic in contemporary educational research.

Recent studies suggest that learning engagement is associated with both psychological and technological factors, particularly in AI-supported learning environments. Among psychological factors, hope and growth mindset have attracted increasing scholarly attention because they represent two important positive psychological resources related to students' motivation, persistence, and learning behaviors. According to Hope Theory (Snyder, 2002), hope refers to individuals' perceived capability to identify pathways toward desired goals and sustain motivation to pursue those goals. Growth mindset, in contrast, reflects the belief that abilities and competencies can be developed through effort, learning, and effective strategies (Dweck, 2006). Although conceptually distinct, both constructs are associated with students' adaptive learning experiences and engagement (Azila-Gbettor et al., 2021; Feldman and Kubota, 2015). Recent studies have further reported significant relationships among hope, growth mindset, and learning engagement in higher education and AI-supported learning contexts (Ghbari et al., 2025; Zhai and Li, 2025). Zhang et al. (2025)found that growth mindset moderated the hope-engagement relationship, while Zhai and Li (2025), drawing on Self-Determination Theory, confirmed that growth mindset—alongside resilience and self-efficacy—facilitated student engagement in AI-based learning environments. Nevertheless, existing studies have primarily examined hope and growth mindset independently, leaving a limited understanding of how these psychological resources may jointly relate to learning engagement in technology-enhanced learning environments.

Alongside psychological factors, technological factors have become increasingly relevant to students' learning experiences. The rapid integration of generative artificial intelligence (GenAI) technologies into higher education has transformed the ways students access information, solve problems, and engage in learning activities (Kasneci et al., 2023; Tlili et al., 2023). Consequently, students' acceptance of GenAI technologies has emerged as an important factor associated with their learning experiences and educational outcomes. Drawing on the Technology Acceptance Model (1989), students who perceive educational technologies as useful and beneficial are more likely to incorporate them into their learning processes. Previous studies have reported positive associations between technology acceptance, self-regulated learning, learning motivation, and learning engagement in AI-supported environments (An et al., 2024; Hardini et al., 2025; Yang, 2025).

However, despite increasing attention to technology acceptance in educational settings, relatively little research has examined its psychological antecedents. In particular, limited evidence exists regarding how positive psychological resources may be associated with students' acceptance of GenAI technologies. From the perspective of Hope Theory, students with higher levels of hope tend to perceive multiple pathways toward goal attainment and actively seek resources that support their academic pursuits (Snyder, 2002). Within AI-supported learning environments, GenAI tools can be regarded as learning resources that provide academic support, feedback, and information access. Therefore, students' hope may be associated with their willingness to accept and utilize GenAI technologies, which may, in turn, be related to learning engagement. Examining this psychological-technological linkage may contribute to a more comprehensive understanding of learning engagement in contemporary educational contexts.

To address these gaps, the present study proposes a conditional direct effect model integrating hope, generative AI acceptance, growth mindset, and learning engagement. Specifically, this study examines whether generative AI acceptance mediates the relationship between hope and learning engagement and whether growth mindset moderates the association between hope and learning engagement. By integrating psychological and technological perspectives within a single framework, this study seeks to extend current understanding of the factors associated with learning engagement in the era of generative AI.

## 2 Literature review

### 2.1 Relationship between hope and learning engagement

Hope is a core construct in positive psychology. Snyder et al. (1991) described hope as a goal-directed cognitive-motivational state that consists of two types of thinking: Pathways thinking and Agency thinking. Pathways thinking refers to individuals' perceived ability to identify routes toward desired goals, whereas agency thinking reflects their motivation to pursue those goals. Previous studies have suggested that hope is associated with persistence and adaptive goal-directed behavior when individuals encounter challenges (Gallagher, 2023; Snyder, 2002).

Learning engagement encompasses students' behavioral effort, emotional connection, and cognitive investment in learning activities (Fredricks et al., 2004). Existing research has consistently reported positive associations between hope and learning engagement. For example, Gallagher et al. (2017) found that hope was associated with engagement trajectories throughout college, while Azila-Gbettor et al. (2021) reported that hope was positively related to university students' engagement. More recent studies have also confirmed the positive relationship between hope and engagement in different educational contexts (Ghbari et al., 2025; Zhang et al., 2025).

In AI-supported learning environments, hope may be particularly relevant because students increasingly rely on technological resources to support learning. From the perspective of Hope Theory (Snyder, 2002), students with higher levels of hope are more likely to seek alternative pathways and resources that facilitate goal attainment. Pathways thinking enables students to recognize generative AI as an instrument that expands available paths toward their learning objectives, and agency thinking fuels the willingness to adopt and persist in using such tools. These two dimensions jointly shape students' generative AI acceptance. Generative AI technologies may therefore be viewed as valuable learning resources that support academic goals. Consequently, hope may be associated not only with learning engagement but also with students' acceptance of generative AI technologies. Although previous studies have confirmed the importance of hope in learning engagement, limited research has examined whether generative AI acceptance represents a mechanism linking hope to learning engagement.

### 2.2 Mediating effect of generative AI acceptance

With the rapid development of generative AI in higher education, increasing attention has been paid to students' acceptance of AI technologies and their learning-related outcomes (Baytak, 2023). Existing research has reported positive relationships between technology acceptance and learning behaviors. Kanont et al. (2024) found that positive attitudes toward generative AI were associated with stronger intentions to use AI for learning purposes. Similarly, Saihi et al. (2024) reported that generative AI acceptance was positively related to learning engagement. Studies have also emphasized the importance of learners' psychological characteristics, including trust, self-efficacy, and anxiety, as antecedents of AI acceptance (Cengiz and Peker, 2025; Montag et al., 2023). Higher acceptance of generative AI may be associated with more active learning participation and engagement (An et al., 2022, 2024).

From a psychological–technological perspective, hope and generative AI acceptance represent two complementary resources that may be associated with learning engagement. Hope reflects students' motivation and perceived capability to pursue academic goals, whereas generative AI acceptance reflects their willingness to utilize technology-based resources to support these goals. According to Hope Theory, individuals with higher levels of hope are more likely to identify pathways and resources that facilitate goal attainment (Snyder, 2002). In AI-supported learning environments, generative AI technologies may be perceived as valuable learning resources because they provide adaptive feedback, information access, and personalized support. Consequently, students with higher levels of hope may be more inclined to view generative AI as a useful pathway for learning and report greater acceptance of these technologies.

Furthermore, the Theory of Planned Behavior suggests that positive attitudes toward a behavior are associated with stronger behavioral intentions (Ajzen, 1991). Students who report higher acceptance of generative AI may therefore be more willing to incorporate these technologies into their learning activities, which is associated with greater learning engagement. Unlike traditional educational technologies, generative AI offers interactive, adaptive, and content-generating support, making students' acceptance of these tools particularly relevant in contemporary learning environments. Therefore, generative AI acceptance may represent a psychological–technological mechanism through which hope is associated with learning engagement. However, empirical evidence examining this indirect association remains limited, highlighting the need for further investigation.

### 2.3 Moderating effect of growth mindset

Growth mindset, based on Dweck's theory, refers to the belief system held by individuals that their abilities can be continuously improved by hard work and the process of learning (Dweck, 2006). There is substantial evidence to show that the concept of growth mindset improves the motivation to learn, self-efficacy, and persistence to learn (Jeon, 2023; Rhew et al., 2018). In the context of studying engagement in the process of learning, the concept of growth mindset has been recognized to have the potential to be the mediator and the moderator in the process (An, 2022; Wu and Lee, 2024). The relevance of a growth mindset is increasingly apparent within a postsecondary and AI-supported learning context. Current studies have explored AI-supported learning and have made apparent its pivotal role within a learning engagement process (Zhai and Li, 2025; Zhang et al., 2025).

Beyond its direct association with learning engagement, growth mindset may influence how other psychological resources are associated with engagement. According to the Conservation of Resources (COR) Theory, individuals draw upon multiple personal resources to cope with challenges and maintain adaptive functioning (Hobfoll, 2002). Similarly, Psychological Capital Theory suggests that positive psychological resources often operate jointly rather than independently in shaping learning-related outcomes (Luthans et al., 2007). Hope reflects students' goal-directed motivation and perceived pathways toward academic success (Snyder, 2002), whereas growth mindset reflects beliefs about the malleability of ability and the potential for improvement through effort (Dweck, 2006). Although both constructs have been associated with positive educational outcomes, they may function in complementary rather than purely additive ways.

Students with a stronger growth mindset are more likely to interpret learning difficulties and technological challenges as opportunities for improvement rather than indicators of fixed ability (Burnette et al., 2013; Yeager and Dweck, 2012). Consequently, their engagement may be sustained by their beliefs about learning and development even when other motivational resources are less salient. In contrast, students with a lower growth mindset may rely more heavily on hope as a motivational resource to maintain engagement when encountering setbacks, uncertainty, or challenges in AI-supported learning environments. This perspective is consistent with resource compensation arguments suggesting that the importance of one psychological resource may increase when another resource is relatively weak (Hobfoll, 2002; Xanthopoulou et al., 2007). Therefore, growth mindset may function as a boundary condition that influences the strength of the association between hope and learning engagement. However, empirical evidence regarding the interaction between hope and growth mindset remains limited, particularly in generative AI-supported learning environments. Accordingly, further investigation is warranted.

In summary, although previous studies have examined hope, generative AI acceptance, and growth mindset separately, these variables have rarely been integrated within a single framework. Furthermore, limited evidence exists regarding whether generative AI acceptance serves as a mechanism linking hope to learning engagement and whether growth mindset functions as a boundary condition in this relationship. Therefore, this study proposes a conditional direct effect model in which hope is associated with learning engagement through generative AI acceptance, while growth mindset moderates the relationship between hope and learning engagement. This framework aims to advance understanding of learning engagement in generative AI-supported learning environments.

## 3 Methods

### 3.1 Research model

**Figure 1:**
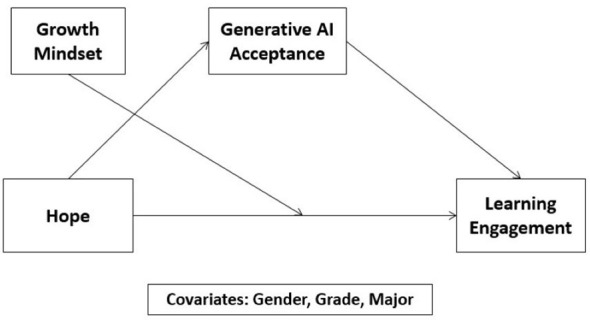
Research model.

To test the proposed model (Figure 1), PROCESS Model 5 (Hayes, 2002) was employed. Model 5 is appropriate when a mediator transmits the association between an independent variable and an outcome variable, while a moderator influences the direct relationship between the independent variable and the outcome variable. In the present study, generative AI acceptance was specified as the mediator linking hope and learning engagement, whereas growth mindset was hypothesized to moderate the direct association between hope and learning engagement. Therefore, PROCESS Model 5 corresponded directly to the proposed conceptual framework and research questions. Previous research has shown that gender and grade may be related to college students' learning engagement. To avoid the influence of control variables such as gender, grade, and major, these factors were controlled in subsequent analyses.

### 3.2 Participants and procedures

This study employed a convenience sampling strategy and recruited university students from 32 provincial-level administrative regions in China through the Wenjuanxing online survey platform. Only currently enrolled university students were eligible to participate. Before participation, students were informed of the study objectives, confidentiality measures, voluntary participation, and their right to withdraw at any stage without penalty. Informed consent was obtained from all participants before data collection.

A total of 622 questionnaires were collected. To ensure data quality, responses with missing values, excessively short completion times, and inattentive response patterns (e.g., straight-lining or identical responses across items) were removed. After data screening, 478 valid questionnaires were retained for subsequent analyses, yielding a valid response rate of 76.8%. The final sample size exceeded the minimum recommendations for covariance-based structural equation modeling and was considered sufficient for testing the proposed research model.

Among the respondents, 199 were male (41.6%), and 279 were female (58.4%). In terms of grade level, 28.7% were first-year students, 37% were second-year students, 25.7% were third-year students, and 8.6% were fourth-year students. The majority of students revealed that 70.7% were in the humanities and social sciences, 29.3% were in the natural sciences. According to the frequency of using generative AI, 4.8% students never use, 25.3% students rarely use, 47.3% students use generative AI sometimes, 18.8% students often use, and 3.8% students always use.

### 3.3 Measuring instruments

#### 3.3.1 Hope

Originating from the scale designed by Snyder, 2002, this scale consists of 8 items in total, with items such as “I can think of many ways to get out of a jam.” It's a 5-point Likert scale ranging from “Strongly disagree” to “Strongly agree.” An increasing score represents stronger hope. In this study, the Cronbach's α was 0.931, and the fitting of the validation factor analysis was adequate [χ2/df = 3.115, CFI = 0.988, TLI = 0.980, RMSEA = 0.067, SRMR = 0.020, AVE=0.627, CR=0.930]. All exceeding the recommended thresholds, these indices exceeded the recommended thresholds (e.g., CFI and TLI > 0.90; RMSEA <0.08; CR > 0.7; AVE > 0.5), confirming that the measurement model represents a robust fit for the empirical data.

#### 3.3.2 Generative AI acceptance

Generative AI acceptance was measured using a revised research questionnaire by Zhang (2023) of the Technology Acceptance Model (Davis, 1989). This scale is designed to measure generative AI acceptance, with items such as “Generative AI tools help me save time when searching for information,” and consists of a total of 17 questions. The measurement was conducted using a 5-point Likert scale, with higher scores indicating a higher level of generative AI acceptance. In this study, the Cronbach's α was 0.906. To ensure the construct validity of the scale in the current study, a 17-item initial pool was subjected to a refinement process. After removing five items with low factor loadings (item6, item8, item14) or high cross-loadings (item7, item 12), the final 12-item model demonstrated a superior fit to the data [χ2/df = 3.808, CFI = 0.964, TLI = 0.952, RMSEA= 0.077, SRMR = 0.058], for the multidimensional construct of generative AI acceptance, convergent validity was assessed at the first-order factor level. The composite reliability (CR) values ranged from 0.867 to 0.909, and the average variance extracted (AVE) values ranged from 0.619 to 0.769.

#### 3.3.3 Growth mindset

The growth mindset (GM) scale used in this study was developed by Dweck (2006). This scale is designed to measure beliefs about changes in intelligence and personality, consisting of four positively worded items and four negatively worded (reverse-coded) items, such as “Intelligence (IQ) is innate and unchangeable (inverted items) and “People have different personalities, but everyone can change their personality.” The measurements were performed on a 5-point Likert scale, with higher scores indicating a higher growth mindset. In this study, the Cronbach's α was 0.884, and the fitting of the validation factor analysis was adequate [χ2/df = 2.339, CFI = 0.991, TLI = 0.982, RMSEA = 0.053, SRMR = 0.031, AVE=0.501, CR=0.886]. All exceeding the recommended thresholds, these indices exceeded the recommended thresholds (e.g., CFI and TLI > 0.90; RMSEA <0.08; CR > 0.7; AVE > 0.5), confirming that the measurement model represents a robust fit for the empirical data.

#### 3.3.4 Learning engagement

The learning engagement (LE) scale in this study used the scale developed by Lam et al. (2014). This scale is designed to measure college students' learning engagement, with items such as “At school, I strive to perform well” and “When studying, I decide which key points to focus on rather than reading broadly,” and consists of a total of 15 questions. The measurement was conducted using a 5-point Likert scale, with higher scores indicating higher levels of learning engagement. In this study, the Cronbach's alpha value was 0.954. Based on the CFA results, four items were iteratively deleted, including high cross-loadings (item1, item2, item3) and negative loading (item7) to improve the model fit and ensure discriminant validity. The validation factor analysis (CFA) showed the acceptable fitting index of the modified scale, the final 11-item measurement model demonstrated a superior fit to the data [χ2/df = 3.931, CFI = 0.978, TLI = 0.967, RMSEA = 0.078, SRMR = 0.027, AVE=0.653, CR=0.953].

Following established psychometric practices, items with low factor loadings, substantial cross-loadings, or negative loadings were removed during CFA model refinement. Importantly, all retained scales continued to represent the theoretical dimensions of the original instruments, thereby preserving content validity while improving construct validity and model fit.

### 3.4 Data analysis

This study used SPSS 26.0 for data analysis, including descriptive statistics and correlation analysis of variables. Additionally, the Unmeasured Latent Method Construct test was applied to check for common method bias. AMOS 24.0 was used to test the model fit of variables and the research model. Finally, the SPSS PROCESS macro model No.5 was undertaken to show the conditional direct path among the variables. In addition, to analyze the conditional direct effect, prior to the analysis, continuous variables were mean-centered, the confidence level of the output confidence interval was 95%, and the number of bootstrap samples for the percentile bootstrap confidence interval was 5,000.

To further examine the potential influence of common method bias, the unmeasured latent method construct (ULMC) approach was employed (Podsakoff et al., 2003). A latent method factor was added to the measurement model, with all observed indicators loading onto both their theoretical constructs and the method factor. The results indicated that the model fit improved after controlling for the method factor, as evidenced by a reduction in RMSEA from 0.065 to 0.061, and CFI increased from 0.909 to 0.920. How, the changes in fit indices between the two models were relatively small (ΔRMSEA = 0.004, ΔCFI = 0.011) and remained below the commonly recommended thresholds (CFI/TLI <0.05; RMSEA <0.015). Therefore, common method bias was unlikely to have substantially affected the findings of the present study.

The model fit of “theoretical model” was excellent (χ2/df = 3.003, CFI = 0.909, IFI = 0.910, RMSEA = 0.065, SRMR = 0.059). To assess discriminant validity, this study employed the Heterotrait-Monotrait (HTMT) ratio of correlations, which is considered more robust than the Fornell-Larcker criterion in detecting a lack of discriminant validity. HTMT values should be below the conservative threshold of 0.85. As shown in Table 1, all HTMT ratios were below 0.85. It provides evidence of discriminant validity between the constructs.

**Table 1:** HTMT Results for discriminant validity.

Variables	Hope	GAA	LE	GM
Hope				
GAA	0.405			
LE	0.712	0.518		
GM	0.462	0.517	0.571	

GAA: Generative AI Acceptance; GM: Growth Mindset; LE: Learning Engagement.

## 4 Results

### 4.1 Descriptive statistics and correlations

The results of the Pearson correlation analysis are presented in Table 2. Hope showed a significant positive correlation with GAA (*r* = 0.372, *p*< *0.0*1), LE (*r* = 0.671, *p*< *0.0*1) and GM (r = 0.417, *p*< *0.0*1). GAA was positively correlated with LE (r = 0.482, *p*< *0.0*1), and GM (r = 0.458, *p*< *0.0*1). LE showed a significant correlated with GM (r = 0.517, *p*< *0.0*1).

**Table 2:** Results of correlation and descriptive statistics analysis.

Variables	Hope	GAA	LE	GM
Hope	1			
GAA	0.372^**^	1		
LE	0.671^**^	0.482^**^	1	
GM	0.417^**^	0.458^**^	0.517^**^	1
M	3.58	3.609	3.737	3.423
SD	0.675	0.537	0.634	0.63
Skewness	0.346	0.539	0.04	0.408
kurtosis	−0.075	0.409	0.381	0.986

^**^p <0.01; GAA, Generative AI Acceptance; GM, Growth Mindset; LE, Learning Engagement.

To assess the potential for multicollinearity, with LE as the dependent variable and hope, GAA, and GM as independent variables, the Variance Inflation Factor (VIF) was calculated for all predictor variables. The results showed that the VIF values ranged from 1.302 to 1.493, which were well below the conservative threshold of 5 (Hair et al., 2022). Therefore, multicollinearity was not a significant concern in this study.

As a result of the descriptive statistical analysis, the mean values of all four major variables exceeded the median value of 3 points, with learning engagement showing the highest mean (M=3.737) among them. The distributional properties of the variables were examined through skewness and kurtosis statistics. All values fell within acceptable ranges (|skewness| <2 and |kurtosis| <7), indicating that the data approximated normality and were suitable for subsequent analyses (Curran et al., 1996).

### 4.2 Conditional direct effect

**Figure 2:**
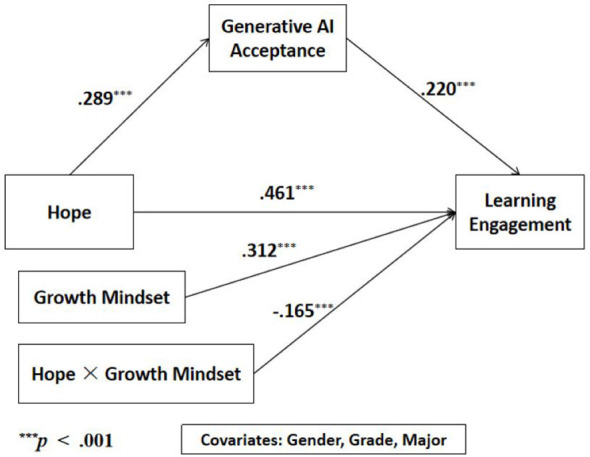
Statistical model of conditional direct effect.

**Figure 3:**
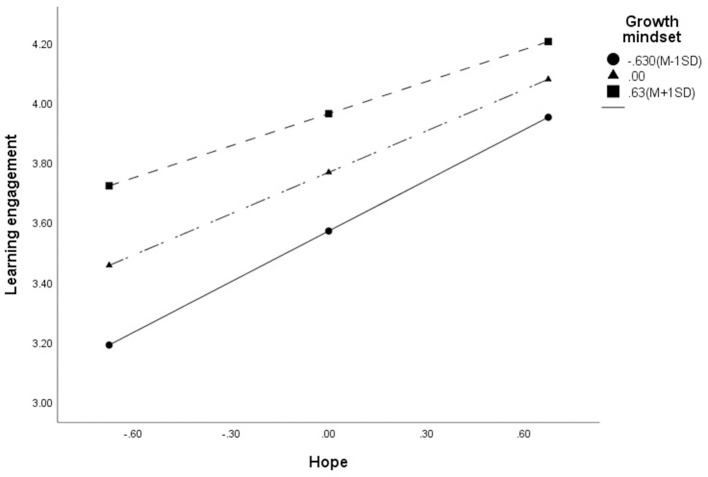
Moderating effect of growth mindset.

To examine whether growth mindset moderates the relationship between hope and learning engagement, that is, the conditional direct effect of growth mindset, the analysis was performed by applying PROCESS macro's Model 5 proposed by Hayes (2002). The analysis results are presented in Figures 2, 3, Tables 3, 4.

**Table 3:** Results of conditional direct effect analysis.

Variables		Mediating variable model			Dependent variable model		
		(DV: GAA)			(DV: LE)		
		Co-effect	*SE*	*t*-value	Co-effect	SE	*t*-value
Constant		3.539	0.149	23.803^***^	2.821	0.196	14.365^***^
ID	Hope	0.289	0.034	8.522^***^	0.461	0.032	14.244^***^
Mediating variable	GAA				0.220	0.042	5.291^***^
Moderator	GM				0.312	0.041	7.527^***^
Interaction	Hope^*^GM				−0.165	0.038	−4.309^***^
Highest order test	*R*^2^ change				0.017		
	*F*				18.569^***^		
Covariates	Gender	−0.021	0.050	−0.418	0.070	0.042	1.663
	Grade	0.063	0.026	2.398^*^	−0.040	0.022	−1.790
	Major	−0.025	0.057	−0.445	0.098	0.048	2.036^*^
Model summary	*R* ^2^	0.152			0.573		
	F	21.200^***^			90.217^***^		
Conditional effects of hope on values of growth mindset							
**GM**	**Effect(B)**	***SE***			***t*** **-value**	**LLCI**	**ULCI**
−0.630(M-1SD)	0.564	0.038			14.696	0.489	0.640
0.000 (M)	0.461	0.032			14.244	0.397	0.524
0.630 (M+1SD)	0.357	0.042			8.466	0.274	0.440
Johnson-Neyman's conditional effect Significance region							
−2.423	0.860	0.095			9.040^***^	0.673	1.047
1.577	0.201	0.071			2.821^**^	0.061	0.340

^*^p <0.05, ^**^p <0.01, ^***^p <0.001, ID, Independent variable; DV, Dependent variable;GAA, Generative AI Acceptance; GM, Growth Mindset; LE, Learning Engagement.

**Table 4:** Analysis of the direct and indirect effects of hope on learning engagement.

Growth mindset	Effect (B)	Boot SE	Boot LLCI	Boot ULCI
Conditional direct effects (hope→learning engagement)				
−0.630 (M-1SD)	0.564	0.038	0.489	0.640
0.000 (M)	0.461	0.032	0.397	0.524
0.630 (M+1SD)	0.357	0.042	0.274	0.440
	Effect (B)	Boot SE	Boot LLCI	Boot ULCI
Indirect effect (hope→generative AI acceptance→learning engagement)				
	0.064	0.019	0.031	0.105

In the mediating variable model, hope positively affected the GAA (*B* = 0.289, *p* <0.001). In the dependent variable model, hope had a significant positive effect on LE (*B* = 0.461, *p* <0.001), and GAA had a significant positive effect on LE (*B* = 0.220, *p* <0.001). As hope had a significant effect on GAA, and GAA had a significant effect on LE, GAA mediated the relationship between hope and LE.

The interaction term between hope and GM had a significant effect on LE (*B*= −0.165, *p* <0.001). The R^2^ change amount (High order test) according to the addition of the interaction term was significant (0.017, *p*< *0.0*01). Therefore, GM moderated the effect of hope on LE. Since the moderating effect was significant, the conditional effect of hope according to the three conditions of GM (M, M ± SD) was analyzed. As a result of the analysis, the conditional effect was significant in all three conditions of GM, but the conditional effect decreased as GM increased from M-SD to M+SD. In other words, as GM increased, the positive effect of hope on LE decreased. As a result of confirming the significance level area of the conditional effects of Johnson- Neyman, the conditional effect was significant in the entire area.

The graph shows the conditioning effect of growth mindset on the hope–learning engagement association as shown in [Figure 3]. Under the three conditions of growth mindset (M, M ± SD), when hope increased, learning engagement increased, but when the growth mindset was M–SD, the increasing slope was steeper, whereas when the growth mindset was M+SD, the slope was relatively flatter compared to when it was M–SD. In other words, even if hope increased equally, learning engagement increased more sharply when the growth mindset was M–SD, but the degree of increase was attenuated when the growth mindset was M + SD—a pattern consistent with resource substitution rather than amplification.

The conditional direct effects and the indirect effect of generative AI acceptance are presented in Table 4. It can be seen that the conditional direct effects of hope on learning engagement under the condition of growth mindset were all significant, and notably, the effect of hope on learning engagement DECREASED as growth mindset increased from M–SD to M+SD (from *B* = 0.564 to *B* = 0.357). In addition, the indirect effect of generative AI acceptance was B=0.064, which was significant because the 95% bootstrap confidence interval [0.031,0.105] did not include zero. These results indicate that the effect of hope on learning engagement can be channeled through generative AI acceptance (indirect pathway) and conditioned by growth mindset (moderation of the direct pathway), with growth mindset partially substituting for the motivational function of hope.

## 5 Discussion

This study aims to explore the mechanism by which hope influences university students' learning engagement, and to examine the mediating roles of generative AI acceptance and the moderating role of growth mindset. The results show that there are significant positive correlations among hope and generative AI acceptance, growth mindset, and learning engagement. The direct effect of hope on learning engagement is significant, and the mediation effect of generative AI acceptance is significant. Furthermore, growth mindset significantly moderates the effect of hope on learning engagement, and the conditional direct effect is significant. These findings will be discussed in more detail below.

### 5.1 The relationship between hope and learning engagement

The results of this study show that hope has a significant positive predictive effect on learning engagement, a finding highly consistent with classic theories in positive psychology and educational psychology. According to the hope theory proposed by Snyder et al., hope motivates individuals to maintain sustained effort when facing learning goals and to actively seek alternative paths when encountering difficulties (Snyder, 2002). Numerous empirical studies have shown that hope can significantly enhance students' behavioral, affective, and cognitive engagement (Azila-Gbettor et al., 2021; Gallagher et al., 2017; Marques et al., 2011).

In structural research on learning engagement, Fredricks et al. (2004) pointed out that students‘ learning engagement is not only influenced by external factors such as instructional design, but also depends more deeply on their internal psychological resources. Research in recent years has further confirmed that hope, as a core form of psychological capital, can stably predict levels of learning engagement across different cultural backgrounds and educational stages. For example, Ghbari et al. (2025) found that academic hope is the strongest predictor of academic engagement, and interventions based on hope can enhance students' learning participation.

In the context of AI-supported learning environments, students with higher levels of hope may be more willing to invest effort in learning because they perceive themselves as capable of overcoming obstacles and achieving academic success. Consequently, hope represents an important motivational foundation for fostering learning engagement in contemporary higher education settings.

### 5.2 The mediating effect of generative AI acceptance

The findings suggest that generative AI acceptance partially mediates the relationship between hope and learning engagement. This discovery expands the explanatory boundaries of the Technology Acceptance Model (TAM) in generative AI learning contexts. The classic TAM posits that an individual's perceived usefulness and ease of use of technology are direct antecedents of their technology use behavior (Davis, 1989; Venkatesh and Davis, 2000), while subsequent research has increasingly emphasized the fundamental role of learners' psychological traits in technology acceptance.

Research in educational technology has demonstrated that students' acceptance of generative AI significantly influences their learning engagement. Schindler et al. (2017) found that a positive attitude toward technology enhances students' deep involvement in digital learning environments. Recent studies indicate that AI markedly improves student participation, cognitive skills, digital literacy, and resource utilization efficiency (Hardini et al., 2025; Xia et al., 2025). Notably, the acceptance of generative AI is influenced not only by technical proficiency but also closely linked to students' learning beliefs and positive emotions (Hardini et al., 2025; Yilmaz and Karaoglan Yilmaz, 2023).

The findings of this study reveal that students with higher levels of hope are more likely to view generative AI tools with a positive and open attitude, thus being more willing to use and integrate these tools in their learning process, ultimately increasing their learning engagement. This indicates that the acceptance of generative AI is not simply a technological attitude variable, but rather an important psychological mechanism by which psychological resources such as hope are transformed into learning behavior. This finding responds to current research calls regarding ‘how generative AI affects learning engagement', providing new empirical evidence for understanding the interaction between AI technology and students' psychological factors.

The present findings extend existing literature by identifying a psychological–technological pathway through which hope influences learning engagement. Hope functions as a psychological resource that motivates students to pursue learning goals, whereas generative AI acceptance serves as a technological pathway that enables students to access and utilize AI-supported learning opportunities. This finding supports the view that positive educational outcomes emerge not only from students' internal psychological strengths but also from their willingness to embrace innovative educational technologies.

### 5.3 The moderating role of growth mindset

This study found that growth mindset plays a significant moderating role between hope and learning engagement. Existing research indicates that growth mindset can enhance students' resilience in academically stressful and challenging situations (Yeager and Dweck, 2012). Recent research has begun to incorporate growth mindset into technology-enhanced learning and AI education, finding that it significantly predicts student engagement and promotes positive use of new technologies (Zhai and Li, 2025). Zhang et al. (2025) found that growth mindset moderated the relationship between hope and learning engagement: when students possess a stronger growth mindset, the positive impact of high hope on learning engagement is significantly enhanced.

However, contrary to the commonly assumed strengthening effect, the present study found that growth mindset weakened rather than strengthened the positive association between hope and learning engagement. Specifically, the positive association between hope and learning engagement was stronger among students with lower levels of growth mindset and weaker among those with higher levels of growth mindset. Although this finding differs from studies emphasizing the beneficial role of growth mindset in promoting engagement and persistence (Dweck, 2006; Yeager and Dweck, 2012), it can be interpreted through the lens of the Conservation of Resources (COR) theory (Hobfoll, 2002). COR theory suggests that individuals possess multiple psychological resources that jointly support adaptation and goal attainment. Hope may be viewed as a motivational resource that energizes goal pursuit, whereas growth mindset represents a cognitive resource reflecting beliefs about the malleability of abilities. Students with stronger growth mindset beliefs may remain engaged because they view challenges as opportunities for learning and improvement, thereby reducing their reliance on hope as a motivational resource. In contrast, students with a lower growth mindset may depend more heavily on hope to sustain engagement when encountering academic or technological challenges. This compensatory pattern may be particularly relevant in generative AI-supported learning environments, where learners frequently face uncertainty, novel learning demands, and rapidly evolving technologies.

Overall, the present findings contribute to the literature by providing additional evidence regarding the interplay between hope and growth mindset from a resource-based perspective. Rather than operating in a purely additive manner, different psychological resources may function in complementary ways when associated with learning engagement. These findings highlight the importance of considering interactions among multiple psychological resources when examining student engagement in technology-enhanced learning environments.

## 6 Conclusion, limitations, and future studies

This study examined the relationships among hope, generative AI acceptance, growth mindset, and learning engagement among university students in AI-supported learning environments. Drawing on Hope Theory, Technology Acceptance Theory, and Conservation of Resources Theory, a conditional direct effect model was developed to explore how psychological and technological factors are associated with learning engagement.

The findings indicated that hope was positively associated with learning engagement. In addition, generative AI acceptance was found to mediate the relationship between hope and learning engagement, suggesting that higher levels of hope were associated with greater acceptance of generative AI technologies, which was in turn associated with higher levels of learning engagement. Furthermore, growth mindset significantly moderated the direct association between hope and learning engagement. Specifically, the positive association between hope and learning engagement was stronger among students with lower levels of growth mindset and weaker among those with higher levels of growth mindset. From a Conservation of Resources perspective, this pattern may reflect a resource substitution mechanism in which growth mindset serves as an alternative psychological resource that reduces reliance on hope for maintaining engagement.

This study contributes to the literature in several ways. First, it provides additional evidence regarding the role of generative AI acceptance as a psychological–technological mechanism associated with the relationship between hope and learning engagement. Second, it enriches current understanding of learning engagement in AI-supported learning environments by integrating psychological resources and technology acceptance within a single framework. Third, the findings offer a resource-based interpretation of how hope and growth mindset may jointly relate to learning engagement.

From a practical perspective, the findings suggest that higher education institutions may benefit from simultaneously supporting students' psychological resources and their readiness to engage with generative AI technologies. Developing students' goal-directed thinking, encouraging adaptive beliefs about learning and ability development, and fostering positive attitudes toward AI-supported learning tools may be associated with higher levels of learning engagement.

Several limitations should be acknowledged. First, this study employed a cross-sectional questionnaire design, which limited the rigor of causal inferences. Future research could utilize longitudinal designs or experimental methods to more precisely examine the causal relationships and developmental trajectories between hope, acceptance of generative AI, and learning engagement. Second, the study primarily relied on self-report questionnaire data, which may be susceptible to common methodological bias. Future research could incorporate multi-source data, such as learning logs, platform behavior data, or teacher evaluations, to enhance the robustness of the findings. Third, using acceptance of generative AI as a single mediating variable does not exhaustively cover all potential psychological mechanisms. Future research could construct multi-mediation or chain-mediation models to more comprehensively reveal the intrinsic mechanisms by which psychological resources influence learning engagement. Further validation of the study model using cross-cultural samples could enhance the external validity of the research findings.

## Data Availability

The original contributions presented in the study are included in the article/supplementary material, further inquiries can be directed to the corresponding author.

## References

[B1] Ajzen I. (1991). The theory of planned behavior. Organ. Behav. Hum. Decision Process. Theor. Cogn. Self Reg. 50, 179–211. doi: 10.1016/0749-5978(91)90020-T

[B2] An F. Xi L. Yu J. (2024). The relationship between technology acceptance and self-regulated learning: The mediation roles of intrinsic motivation and learning engagement. Educ. Inf. Technol. 29, 2605–2623. doi: 10.1007/s10639-023-11959-3 37361728 PMC10256961

[B3] An F. Yu J. Xi L. (2022). Relationship between perceived teacher support and learning engagement among adolescents: Mediation role of technology acceptance and learning motivation. Front. Psychol. 13:992464. doi: 10.3389/fpsyg.2022.992464 36248506 PMC9562933

[B4] An N. (2022). The relationship between teachers' autonomy support and middle school students' learning engagement: the mediation effect of growth mindset and intrinsic learning motivation. Adv. Soc. Sci. 11, 771–778. doi: 10.12677/ASS.2022.113109

[B5] Azila-Gbettor E. M. Mensah C. Atatsi E. A. Abiemo M. K. (2021). Predicting students' engagement from hope and mindfulness. J. Appl. Res. High. Educ. 14, 1355–1370. doi: 10.1108/JARHE-02-2021-0068

[B6] Baytak A. (2023). The acceptance and diffusion of generative artificial intelligence in education: a literature review. Curr. Perspect. Educ. Res. 6, 7–18. doi: 10.46303/cuper.2023.2

[B7] Burnette J. L. O'Boyle E. H. VanEpps E. M. Pollack J. M. Finkel E. J. (2013). Mind-sets matter: a meta-analytic review of implicit theories and self-regulation. Psychol. Bull. 139, 655–701. doi: 10.1037/a0029531 22866678

[B8] Cengiz S. Peker A. (2025). Generative artificial intelligence acceptance and artificial intelligence anxiety among university students: the sequential mediating role of attitudes toward artificial intelligence and literacy. Curr. Psychol. 44, 7991–8000. doi: 10.1007/s12144-025-07433-7

[B9] Curran P. J. West S. G. Finch J. F. (1996). The robustness of test statistics to nonnormality and specification error in confirmatory factor analysis. Psychol. Methods 1, 16–29. doi: 10.1037/1082-989X.1.1.16

[B10] Davis F. D. (1989). Perceived usefulness, perceived ease of use, and user acceptance of information technology. MIS Q. 13:319. doi: 10.2307/249008

[B11] Dweck C. (2006). Mindset: The New Psychology of Success. New York, NY: Random House.

[B12] Feldman D. B. Kubota M. (2015). Hope, self-efficacy, optimism, and academic achievement: distinguishing constructs and levels of specificity in predicting college grade-point average. Learn. Individ. Differ. 37, 210–216. doi: 10.1016/j.lindif.2014.11.022

[B13] Fredricks J. A. Blumenfeld P. C. Paris A. H. (2004). School engagement: potential of the concept, state of the evidence. Rev. Educ. Res. 74, 59–109. doi: 10.3102/00346543074001059

[B14] Gallagher M. W. (2023). The scientific status of the psychology of hope. Curr. Opin. Psychol. 53:101684. doi: 10.1016/j.copsyc.2023.101684 37659285

[B15] Gallagher M. W. Marques S. C. Lopez S. J. (2017). Hope and the academic trajectory of college students. J. Happiness Stud. 18, 341–352. doi: 10.1007/s10902-016-9727-z

[B16] Ghbari T. A. Ghazal M. M. A. Al-Smadi R. T. (2025). Deliberative vs. Implemental mindset and academic hope as predictors of academic engagement among university students. Electron. J. Res. Educ. Psychol. 23, 1–26. doi: 10.25115/ejrep.v23i65.9616

[B17] Hair J. F. Babin B. J. Anderson R. E. Black W. C. (2022). Multivariate Data Analysis. Boston, MA: Cengage Learning.

[B18] Hardini M. Hetilaniar H. Girsang S. E. E. Putra S. N. W. Hikam I. N. (2025). Advancing higher education: longitudinal study on AI integration and its impact on learning. IJCITSM 5, 23–30. doi: 10.34306/ijcitsm.v5i1.185

[B19] Hayes A. F. (2002). Introduction to Mediation, Moderation, and Conditional Process Analysis, 3^*rd*^ *Edn a Regression-Based Approach*. New York, NY: Guilford Press.

[B20] Hobfoll S. E. (2002). Social and psychological resources and adaptation. Rev. Gen. Psychol. 6, 307–324. doi: 10.1037/1089-2680.6.4.307

[B21] Jeon H. (2023). The relationship between the growth mindset and academic adaptation among university students: the serial mediating effect of grit and career adaptation. J. Pract. Eng. Educ. 15, 615–624. doi: 10.14702/JPEE.2023.615

[B22] Kanont K. Pingmuang P. Simasathien T. Wisnuwong S. Wiwatsiripong B. Poonpirome K. . (2024). Generative-AI, a learning assistant? Factors influencing higher-ed students' technology acceptance. Electron. J. E-Learn. 22, 18–33. doi: 10.34190/ejel.22.6.3196

[B23] Kasneci E. Sessler K. Küchemann S. Bannert M. Dementieva D. Fischer F. . (2023). ChatGPT for good? On opportunities and challenges of large language models for education. Learn. Individ. Diff. 103:102274. doi: 10.1016/j.lindif.2023.102274

[B24] Lam S. Jimerson S. Kikas E. Cefai C. Veiga F. H. Nelson B. . (2012). Do girls and boys perceive themselves as equally engaged in school? The results of an international study from 12 countries. J. Sch. Psychol. 50, 77–94. doi: 10.1016/j.jsp.2011.07.004 22386079

[B25] Lam S. Jimerson S. Wong B. P. H. Kikas E. Shin H. Veiga F. H. . (2014). Understanding and measuring student engagement in school: the results of an international study from 12 countries. Sch. Psychol. Q. 29, 213–232. doi: 10.1037/spq0000057 24933218

[B26] Luthans F. Youssef-Morgan C. Avolio B. (2007). Psychological Capital: Developing the Human Competitive Edge. (New York, NY: Oxford University Press), 256. doi: 10.1093/acprof:oso/9780195187526.001.0001

[B27] Marques S. C. Lopez S. J. Pais-Ribeiro J. L. (2011). “Building hope for the future”: a program to foster strengths in middle-school students. J. Happiness Stud. 12, 139–152. doi: 10.1007/s10902-009-9180-3

[B28] Montag C. Kraus J. Baumann M. Rozgonjuk D. (2023). The propensity to trust in (automated) technology mediates the links between technology self-efficacy and fear and acceptance of artificial intelligence. Comput. Hum. Behav. Rep. 11:100315. doi: 10.1016/j.chbr.2023.100315

[B29] Podsakoff P. M. MacKenzie S. B. Lee J.-Y. Podsakoff N. P. (2003). Common method biases in behavioral research: a critical review of the literature and recommended remedies. J. Appl. Psychol. 88, 879–903. doi: 10.1037/0021-9010.88.5.879 14516251

[B30] Rhew E. Piro J. S. Goolkasian P. Cosentino P. (2018). The effects of a growth mindset on self-efficacy and motivation. Cogent. Educ. 5:1492337. doi: 10.1080/2331186X.2018.1492337

[B31] Saihi A. Ben-Daya M. Hariga M. As'ad R. (2024). A structural equation modeling analysis of generative AI chatbots adoption among students and educators in higher education. Comput. Educ. Artif. Intell. 7:100274. doi: 10.1016/j.caeai.2024.100274

[B32] Schindler L. A. Burkholder G. J. Morad O. A. Craig M. (2017). Computer-based technology and student engagement: a critical review of the literature. Int. J. Educ. Technol. High. Educ. 14:38. doi: 10.1186/s41239-017-0063-0

[B33] Snyder C. R. (2002). TARGET ARTICLE: Hope theory: rainbows in the mind. Psychol. Inq. 13, 249–275. doi: 10.1207/S15327965PLI1304_01

[B34] Snyder C. R. Harris C. Anderson J. R. Holleran S. A. Irving L. M. Sigmon S. T. . (1991). The will and the ways: Development and validation of an individual-differences measure of hope. J. Pers. Soc. Psychol. 60, 570–585. doi: 10.1037/0022-3514.60.4.570 2037968

[B35] Tlili A. Shehata B. Adarkwah M. A. Bozkurt A. Hickey D. T. Huang R. . (2023). What if the devil is my guardian angel: ChatGPT as a case study of using chatbots in education. Smart Learn. Environ. 10:15. doi: 10.1186/s40561-023-00237-x

[B36] Venkatesh V. Davis F. D. (2000). A theoretical extension of the technology acceptance model: four longitudinal field studies. Manage. Sci. 46, 186–204. doi: 10.1287/mnsc.46.2.186.11926

[B37] Wang M.-T. Fredricks J. Ye F. Hofkens T. Linn J. S. (2019). Conceptualization and assessment of adolescents' engagement and disengagement in school. Eur. J. Psychol. Assess. 35, 592–606. doi: 10.1027/1015-5759/a000431

[B38] Wu M. Lee C. S. (2024). Effect of academic burnout on academic self-efficacy of Chinese college students: mediating effect of study engagement and moderated mediation effect of growth mindset. Industry Promot. Res. 9, 231–239. doi: 10.21186/IPR.2024.9.1.231

[B39] Xanthopoulou D. Demerouti E. Schaufeli W. (2007). The role of personal resources in the job demands-resources model. Int. J. Stress Manag. 14, 121–141. doi: 10.1037/1072-5245.14.2.121

[B40] Xia Q. Li W. Yang Y. Weng X. Chiu T K. F. (2025). A systematic review and meta-analysis of the effectiveness of generative artificial intelligence (GenAI) on students' motivation and engagement. Comput. Educ. Artif. Intell. 9:100455. doi: 10.1016/j.caeai.2025.100455

[B41] Yang H. (2025). Harnessing generative AI: exploring its impact on cognitive engagement, emotional engagement, learning retention, reward sensitivity, and motivation through reinforcement theory. Learn. Motiv. 90:102136. doi: 10.1016/j.lmot.2025.102136

[B42] Yeager D. S. Dweck C. S. (2012). Mindsets that promote resilience: when students believe that personal characteristics can be developed. Educ. Psychol. 47, 302–314. doi: 10.1080/00461520.2012.722805

[B43] Yilmaz R. Karaoglan Yilmaz F. G. (2023). The effect of generative artificial intelligence (AI)-based tool use on students' computational thinking skills, programming self-efficacy, and motivation. Comput. Educ. Artif. Intell. 4, 100147. doi: 10.1016/j.caeai.2023.100147

[B44] Zhai X. Li S. (2025). The roles of growth mindset, resilience, and self-efficacy in student engagement with AI-enhanced Chinese learning: a self-determination theory perspective. Learn. Motiv. 92, 102183. doi: 10.1016/j.lmot.2025.102183

[B45] Zhang C. (2023). A study on college students' willingness to use generative AI Tools—Based on technology acceptance model. Sci. Technol. Commun. 15, 131–135. doi: 10.16607/j.cnki.1674-6708.2023.23.034

[B46] Zhang H. Wang Q. Xu X. Liu S. (2025). Influence of the effort-reward imbalance in college students on learning engagement: the mediating role of hope and the moderating role of growth mindset. Front. Psychol. 16:1650064. doi: 10.3389/fpsyg.2025.1650064 41159176 PMC12554691

